# Automatic procedure for generating symmetry adapted wavefunctions

**DOI:** 10.1186/s13321-017-0193-3

**Published:** 2017-02-02

**Authors:** Marcus Johansson, Valera Veryazov

**Affiliations:** Division of Theoretical Chemistry, Lund University, Naturvetarvägen 14, P.O.B. 124, 221 00 Lund, Sweden

**Keywords:** Molecular symmetry, Wavefunction, Point group, Symmetry adapted

## Abstract

Automatic detection of point groups as well as symmetrisation of molecular geometry and wavefunctions are useful tools in computational quantum chemistry. Algorithms for developing these tools as well as an implementation are presented. The symmetry detection algorithm is a clustering algorithm for symmetry invariant properties, combined with logical deduction of possible symmetry elements using the geometry of sets of symmetrically equivalent atoms. An algorithm for determining the symmetry adapted linear combinations (SALCs) of atomic orbitals is also presented. The SALCs are constructed with the use of projection operators for the irreducible representations, as well as subgroups for determining splitting fields for a canonical basis. The character tables for the point groups are auto generated, and the algorithm is described. Symmetrisation of molecules use a projection into the totally symmetric space, whereas for wavefunctions projection as well and partner function determination and averaging is used. The software has been released as a stand-alone, open source library under the MIT license and integrated into both computational and molecular modelling software.

**Graphical abstract:**
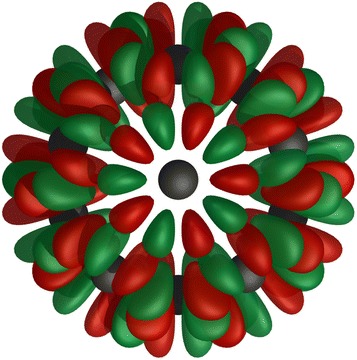
.

.

## Background

In the early days of quantum chemistry, the use of molecular symmetry was motivated by necessity to reduce the size of the problem at hand. Some calculations were only possible due to the ability to split the Hamiltonian into smaller portions, determined by the irreducible representations (irreps) of the symmetry group. In modern times this particular motivation has all but disappeared, since the majority of molecular systems show no symmetry, and computational algorithms are designed to handle large Hamiltonians [1, 2].

Although the use of symmetry can still provide a performance increase, other aspects of using symmetry in quantum mechanics become more important: an ability to obtain the wavefunction with desired symmetry, which does not necessarily match the symmetry of the nuclei. Symmetry breaking of the wavefunction can be caused by external electrostatic or magnetic fields, or observed as anti-ferromagnetic ordering in solids. A symmetry broken solution for a wavefunction can also be caused by the computational method: the most famous example of that kind is Unrestricted Hartree–Fock method, where the symmetry of the electronic wavefunction is lower than the symmetry of nuclei due to the electron spin. Another quite common problem related to mismatch between the symmetry of the nuclei and the symmetry of the wavefunction has a technical nature: many computational codes can not utilise the information about the full point group of the molecule, and uses only a restricted subset (usually an Abelian subgroup). This strategy dramatically reduces the development costs for the quantum chemical software, but it also might lead to non-physical symmetry breaking of the electronic wavefunction. Numerical inaccuracy due to a limited precision or approximations used for the computing the Hamiltonian (e.g. approximate two-electron integrals) might lead to a small split of the degenerate levels, but iterative procedures used in the majority of quantum chemical methods can increase this small difference to a physically wrong description of the system.

Using symmetry when constructing the wavefunction may also place restrictions on the system such as allowed linear combinations or excitations in multi-configurational methods, and depending on the problem one might be interested in the symmetric or non-symmetric solution. If the choice is to not use the full symmetry of the system, it may still be desirable to determine whether or not symmetry breaking has occurred, and if so, what the closest symmetric solution would have looked like.

Automating the procedure for detection as well as the construction on symmetric wavefunctions may have several benefits during a calculation: for large scale systems the symmetry may not be immediately obvious or the input may be slightly symmetry broken causing unexpected results. It may also serve as an aid for research where a more manual approach may be time consuming, such as when experimenting with different basis sets.

There is also the educational aspect of having a tool that can aid in spatial visualisation of molecular symmetry since the ability to internally visualise all symmetry elements of a group may vary between students. Not only can the elements of the group be determined and visualised in an interactive environment, but the symmetry adaptation of orbitals can be shown, providing an immediate visual answer to questions such as: What would it mean to be an eigenfunction to all the operators in this group?

In this article we present a new library for detecting the molecular symmetry and using it for the symmetrisation of wavefunctions. The library, called libmsym, is freely available under an MIT license and can be easily integrated into computational codes as well as into visualisation software.

## Mathematical background

### Symmetry detection

**Fig. 1:**
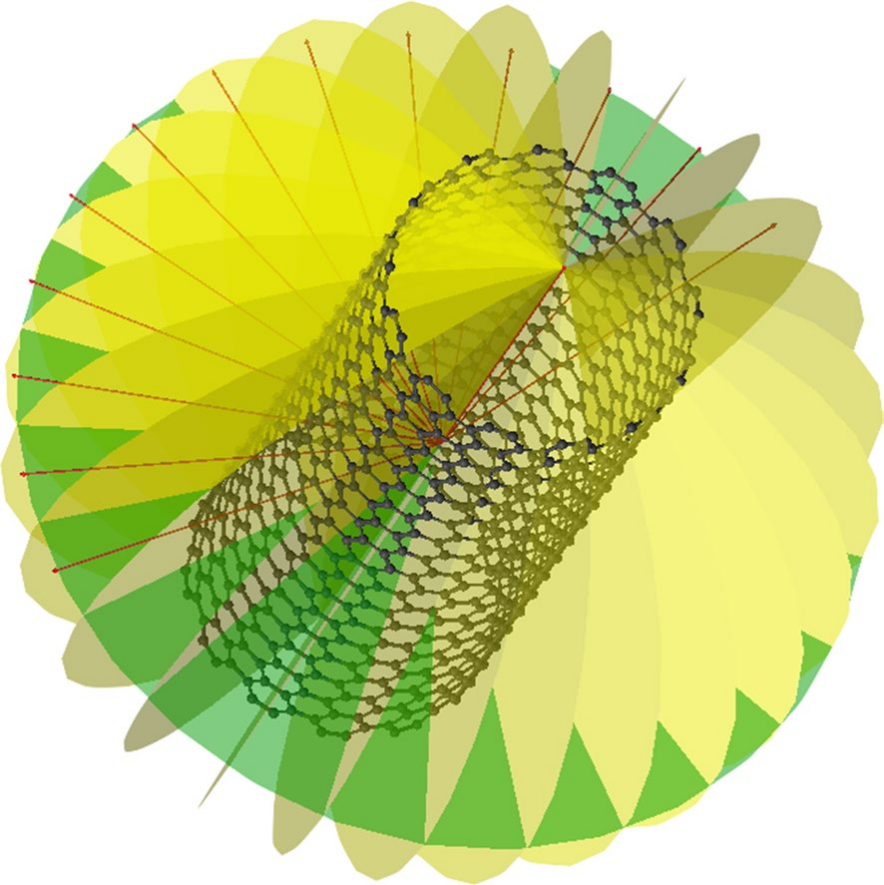
Symmetry elements of nanotube. Symmetry elements of a $D_{13d}$ nanotube produced by libmsym integration in Luscus [11]

In order to symmetrise a molecule or construct SALCs one must first determine the point group. There are several ways to achieve this, the best solution for trivial cases being exhaustive search. Some algorithms make use of graph theory and ideal geometries [3] even in an arbitrary number of dimensions [4], others use euclidean distance matrices [5] and geometry determined by moments of inertia [6, 7]. Some algorithms use exhaustive search in combination with concepts such as equivalence sets [8] to narrow a search field [9]. This approach often restricts [7, 9, 10] the search to some maximum of all possible symmetry operations as there are infinitely many. The approaches described below will make use of many of these concepts, although with some variation. Exhaustive search is employed only in cases where the search field is narrow enough to provide better performance than the calculations required for a more analytical approach, and there is no restriction on the order of a symmetry operation, i.e. finding the symmetry of a molecule belonging to the $D_{47d}$ point group requires no change to the search field from $D_{6d}$. The detected symmetry elements of a nanotube can be seen in Fig. 1 produced by libmsym integration in Luscus [11].

#### Equivalence sets

**Fig. 2:**
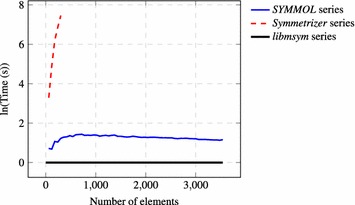
Performance of symmetry detection. Time elapsed for symmetry detection for *SYMMOL* [10] and *Symmetrizer* [9] using a logarithmic scale with *libmsym* as baseline. Note that scaling performance may not have been a priority for the above implementations

Partitioning elements into equivalence sets can be done either prior to or after determination of the point group. On one hand, determining the symmetry elements prior to the equivalence sets leaves little room for error as well as a simple way of defining the equivalence thresholds. On the other hand, equivalence set information can be used to restrict the possible symmetry elements to a point where no search is required. This work takes the latter approach. The partitioning algorithm developed is the most computationally intensive part of determining the point group and has complexity $O(N^2)$, where *N* is the number of elements in a molecule, and a performance comparison can be seen in Fig. 2. The complexity could be reduced further by first applying a sorting algorithm to a linearly scaling property, but that is outside the scope of this work.

Equivalence set partitioning is done using a clustering algorithm for symmetry invariant properties. One such property is a weighted euclidean distance matrix ${\mathbf {D}}$.

Let $\vec {a}_i$ denote the position vector of an atomic nuclei with origin at the centre of mass $a_i \in {\mathbf {A}}$ where ${\mathbf {A}}$ is the set of all atoms in the molecule. We can then define the matrix elements of ${\mathbf {D}}$:

$$\begin{aligned} {\mathbf {D}}_{ij} = \mu _{ij}\left| \vec {d}_{ij}\right| \end{aligned}$$

where $\vec {d}_{ij} = \vec {a}_i - \vec {a}_j$ and $\mu _{ij}$ is the reduced mass of $a_i$ and $a_j$. A permutation of a vector ${\mathbf {D}}_{i*}$ corresponding to an element $a_i$ will produce another vector ${\mathbf {D}}_{j*}$ if $a_i$ and $a_j$ belong to the same equivalence set and the permutation is a symmetry operation. Other properties of ${\mathbf {D}}$ such as the eigenvectors or a euclidean degree partition algorithm [5] can also be used to determine equivalence sets. It does however have the drawback of a memory footprint of order $\left| {\mathbf {A}}\right| ^2$. In order to alleviate this problem statistical properties of the vectors, the mean ($\bar{{\mathbf {D}}}_{i*}$) and length ($\left| {\mathbf {D}}_{i*}\right|$), are used instead. Two additional symmetry invariant properties are used for differentiation, projections onto a unit sphere $\vec {s}_i$ and plane $\vec {p}_i$ scaled by reduced mass:

$$\begin{aligned} \vec {s}_i= & {} \displaystyle \sum _{j} \mu _{ij}\frac{\vec {d}_{ij}}{\left| \vec {d}_{ij}\right| }\\ \vec {p}_i= & {} \displaystyle \sum _{j} \mu _{ij}\left( \vec {a}_j - \frac{\vec {a}_i\cdot \vec {a}_j}{\left| \vec {a}_i\right| ^2}\vec {a}_i\right) \end{aligned}$$

**Fig. 3:**
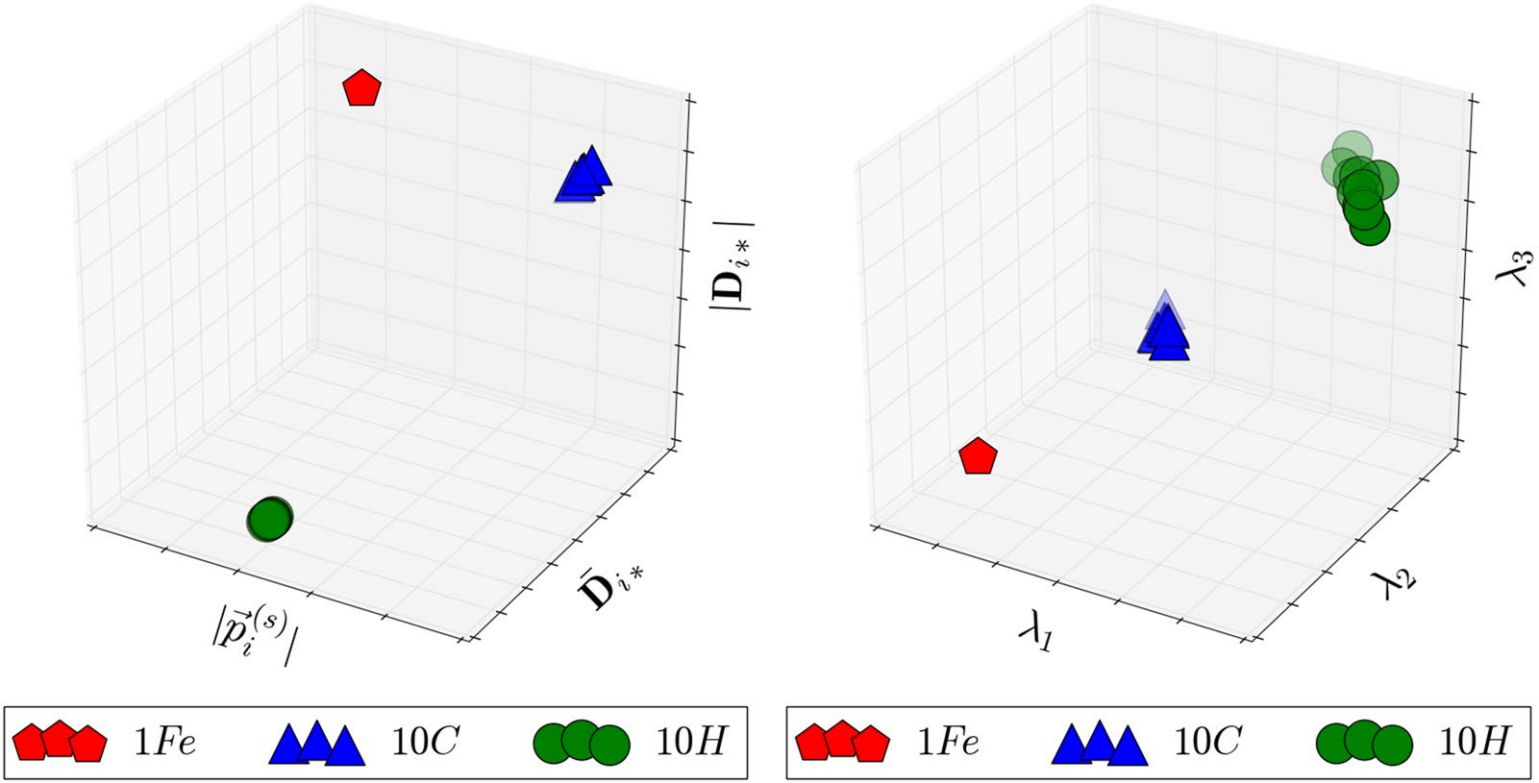
Clustering of symmetry invariant properties. Clustering of symmetry invariant properties in a symmetry broken ferrocene molecule. The plane projection has been omitted due to it being zero in $D_{5d}$, and the limitation of 3D-plots

The relative values of $\bar{{\mathbf {D}}}_{i*}$, $\left| {\mathbf {D}}_{i*}\right|$, $\left| \vec {s}_i\right|$ and $\left| \vec {p}_i\right|$ are used by the clustering algorithm. This idea is general enough to be applicable to any symmetry invariant property, and not necessarily restricted to 3-dimensional space. Figure 3 compares the clustering of the above parameters with the clustering of eigenvalues of the inertial tensor at each point $\vec {a}_i$.

The same partitioning algorithm is applied to the generated subsets, in order to detect differences not seen in the context of the entire molecule. The result is are disjoint sets ${\mathbf {S}}_i^{G} \subseteq {\mathbf {A}}$ of symmetry equivalent nuclei.

#### Symmetry elements

The geometry of the molecule provides some indication of where the symmetry elements can be. The eigenvalues and eigenvectors of the inertial tensor ${\mathbf {I}}_G$ are the principal moments and axes of inertia for the equivalence set:

$$\begin{aligned} {\mathbf {I}}_G = \displaystyle \sum _{a_i \in {\mathbf {A}}} m_{a_i}\left( (\vec {a}_i\cdot \vec {a}_i){\mathbf {E}}-(\vec {a}_i\otimes \vec {a}_i)\right) \end{aligned}$$

where ${\mathbf {E}}$ is the identity matrix in three dimensions. These eigenvalues and eigenvectors are calculated using a Jacobi algorithm for $3\times 3$ matrices.

The moments of inertia indicate the overall geometry, whereas the axes provide a direction. Since all elements in a set are symmetrically equal, the symmetry elements can be determined completely based on the geometry, with the exception for polyhedral groups. Polyhedral groups have triply degenerate principal moments and the axes are therefore arbitrary. Since no directionality is provided, the algorithm used looks for ${\hat{\sigma }}$, ${\hat{C}}_2$ and ${\hat{C}}_4$ operations between equidistant pairs of elements and uses the number of operations found as well as the angles between the axes of symmetry elements to generate the implied operations.

The symmetry elements found in each equivalence set are then intersected so as to make sure they are present in all sets. Since ${\hat{C}}_\infty$ operations imply infinitely many operations, the intersection algorithm generates new operations if the intersect would reduce to a finite set.

#### Determining point groups

To which point group the molecule belongs is determined using standard methods [12], with a few minor changes. Cases where there is a requirement for e.g. $5{\hat{\sigma }}_v$ symmetry elements, are changed to only have the requirement of $1{\hat{\sigma }}_v$, since the remaining elements are implied, and will be generated.

#### Generating symmetric point groups

The symmetry elements need to be as close to symmetrical as possible, and since they are determined from input data, they need to be symmetrised before use. In order to retain the orientation, a transformation matrix for aligning totally symmetric versions of the point group elements with the molecule is used.

The conjugacy class for each operation also needs to be computed. This can be achieved by testing each matrix product in the cartesian basis or directly when generating the point group elements. The former will always generate different classes for operations whose characters are complex, in e.g. the $C_{3}$ point group, whereas the latter allows for the classes to be the same, if linear combinations of the representations are used, as is the case when dealing with real spherical harmonic basis functions.

In addition to the conjugacy class, the orientation of the symmetry operation (horizontal, vertical or dihedral) is also generated, for use in character table generation.

#### Determining subgroups

Subgroups can be used as a splitting field for degenerate irreps, as well as for lowering symmetry if so desired. Although there are ways of computing subgroups during point group generation rather than after the fact, the algorithm employed is a exhaustive search algorithm which uses the permutation cycles of the point group multiplication table. The search space is limited by pre-calculating the number of subgroups prior to searching.

### Symmetry adapted linear combinations (SALCs)

In order to use symmetry during a calculation, such as symmetrisation or restriction of allowed linear combinations, SALCs are required. Using these SALCs the symmetry operations, as well as the Hamiltonian can be represented in block diagonal form. The procedure described below assumes no overlap in the original basis since any overlap will follow the same symmetry. Basis set overlap needs to be taken into account although the required calculations can be done after this procedure.

#### Generating character tables $\chi ^{\Gamma }(R)$

Since there is an infinite number of cyclic and dihedral groups, and the detection algorithm can detect all of them - in theory, not in practice - it makes sense to generate their character tables.

In order to generate the character tables, properties of representations are calculated first and the characters for the symmetry operations are generated based on the operations in combination with the representations. Since the irreps of these groups are 1- or 2-dimensional the scope is greatly limited. The properties of the irreps are generated based on the point group, and are static with the exception of *E*, i.e. 2-dimensional representations.

**Fig. 4:**
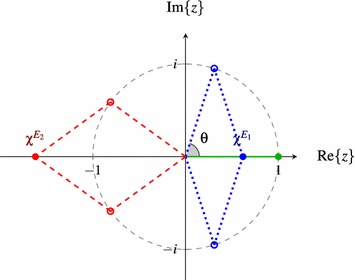
Characters of ${\hat{C}}_5$ Eigenvalues and characters of ${\hat{C}}_5$ in a 2-dimensional irrep, where the characters are marked on the real axis

All characters of 1-dimensional irreps are $\pm 1$, where the sign is determined by properties of the irrep as well whether the symmetry axis is horizontal, vertical or dihedral. The characters of *E*-representations however vary. The character of an operation can be defined in terms of its eigenvalues:

$$\begin{aligned} \chi ^{\Gamma }(R) = \displaystyle \sum _{k=1}^{dim(\Gamma )} \lambda _k \end{aligned}$$

where $\lambda _k$ are the eigenvalues of ${\hat{R}}$ in some representation *D*(*R*). The characters are either $\pm 1$ or come in complex conjugate pairs as illustrated in Fig. 4 using a ${\hat{C}}_5$ operation.

This information is enough to generate the character tables [13]. The real character corresponds to a 1-dimensional irrep and which character corresponds to which *E* is determined by an incremental index, where the character can be calculated using:

$$\begin{aligned} \chi ^{E_j}(C_n^k) = 2\cos \left( jk\frac{2\pi }{n}\right) \end{aligned}$$

where *j* is the index. For improper rotation, the character also depends on how the representation behaves under ${\hat{\sigma }}_h$.

#### Permutations

Since the size of equivalence sets are smaller than the order of the group, the permutation information is generated using exhaustive search. The same algorithm is applied to the group itself in order to produce a canonical non-trivial representation *D*(*G*). Table 1 shows the generated permutation cycles of $D(C_{3v})$.

**Table 1:** $$C_{3v}$$ permutations

${\hat{E}}$	$({\hat{E}})\quad ({\hat{C}}_3)\quad ({\hat{C}}_3^2)\quad ({\hat{\sigma }})\quad ({\hat{\sigma }}')\quad ({\hat{\sigma }}'')$
${\hat{C}}_3$	$({\hat{E}}\;{\hat{C}}_3\;{\hat{C}}_3^2)\quad ({\hat{\sigma }}\;{\hat{\sigma }}'\;{\hat{\sigma }}'')$
${\hat{C}}_3^2$	$({\hat{E}}\;{\hat{C}}_3^2\;{\hat{C}}_3)\quad ({\hat{\sigma }}\;{\hat{\sigma }}''\;{\hat{\sigma }}')$
${\hat{\sigma }}$	$({\hat{E}}\;{\hat{\sigma }})\quad ({\hat{C}}_3\;{\hat{\sigma }}'')\quad ({\hat{C}}_3^2\;{\hat{\sigma }}')$
${\hat{\sigma }}'$	$({\hat{E}}\;{\hat{\sigma }}^{'})\quad ({\hat{C}}_3\;{\hat{\sigma }})\quad ({\hat{C}}_3^2\;{\hat{\sigma }}'')$
${\hat{\sigma }}''$	$({\hat{E}}\;{\hat{\sigma }}'')\quad ({\hat{C}}_3\;{\hat{\sigma }}')\quad ({\hat{C}}_3^2\;{\hat{\sigma }})$

Permutation cycles of $C_{3v}$

**Table 2:** $$C_{3v} \downarrow C_3/C_s$$ subduction

$C_{3v}$	$C_3$	$C_s$
$A_1$	*A*	$A'$
$A_2$	*A*	$A''$
*E*	$e_1 + e_2$	$A' + A''$

Symmetry species of irreducible representations when lowering symmetry from $C_{3v}$ to $C_3$ and $C_s$

#### Characters of spherical harmonics

Let $\{\left| Y_\ell ^m\right\rangle \} = Y$ denote a spherical harmonic basis. In the *O*(3) group, all rotations by the same angle belong to the same conjugacy class and therefore have the same character. For some vector of rotation in an $\left| Y\right|$-dimensional space, the spherical harmonics of order $\ell$ are eigenfunctions to the ${\hat{C}}_n$ operator:

$$\begin{aligned} {\hat{C}}_nY_\ell ^m= \mathrm {e}^{-im\theta }Y_\ell ^m\end{aligned}$$

This means the ${\hat{C}}_n$ in *Y* can be represented by:

$$\begin{aligned} D^{Y}(C_n) = \begin{bmatrix} {\mathrm {e}}^{-i\theta (-\ell )}&0&\cdots&0 \\ 0&{\mathrm {e}}^{-i\theta (-\ell +1)}&\cdots&0 \\ \vdots&\vdots&{\ddots}&\vdots \\ 0&0&\cdots&{\mathrm {e}}^{-i\theta \ell } \end{bmatrix} \end{aligned}$$

where $\theta = \frac{2\pi }{n}$. Since the character is invariant under a similarity transform the character of any ${\hat{C}}_n$ operation is given by:

$$\begin{aligned} \chi ^{Y}(C_n) = \displaystyle \sum _{k=-\ell }^{\ell } \cos (k\theta ) = \frac{\sin \left( \frac{\theta }{2}\left| Y\right| \right) }{\sin \left( \frac{\theta }{2}\right) } \end{aligned}$$

and similarly for ${\hat{S}}_n$ by:

$$\begin{aligned} \chi (S_n) = \frac{\cos \left( \frac{\theta }{2}\left| Y\right| \right) }{\cos \left( \frac{\theta }{2}\right) } \end{aligned}$$

For the remaining symmetry operations $\chi (E) = \left| Y\right|$, $\chi ({\hat{\imath }}) = (-1)^{\ell } \left| Y\right|$ and $\chi (\sigma ) = 1$. These characters will remain the same in all subgroups *G* of *O*(3). The span of the irreps can therefore be calculated without without constructing the matrix representations of the symmetry operations, allowing for more efficient calculations, as well as the providing the benefit of verifying the results.

#### Projection operator ${\hat{P}}^{\Gamma }$

Although projection methods are commonplace when constructing SALCs [12, 14, 15], they are described below in the context of constructing a similarly oriented basis for degenerate representations [14].

Let $\left| \Theta ^{\Gamma }\right\rangle$ denote a SALC transforming as irrep $\Gamma$ in some subspace $V^{\Gamma }$ of the function space *W*. An operator can be constructed which will projects out the *l*-components of the irrep $\Gamma$, and simultaneously acts as a ladder operator yielding the *k*-components [14]:

$$\begin{aligned} {\hat{P}}_{kl}^{\Gamma } = \displaystyle \sum _{m=1}^{m_{\Gamma }}{\left| \Theta _{m,k}^{\Gamma }\right\rangle \left\langle \Theta _{m,l}^{\Gamma }\right| } = \frac{dim(\Gamma )}{\left| G\right| }\displaystyle \sum _{R \in G}{\Gamma _{kl}(R){\hat{R}}} \end{aligned}$$

where $m_{\Gamma }$ is the multiplicity with which $\Gamma$ occurs in *D*(*G*) according to:

$$\begin{aligned} D(R) = \bigoplus _{\Gamma }{m_{\Gamma }\Gamma (R)}, R \in G \end{aligned}$$

This means that ${\hat{P}}_{kl}^{\Gamma }$ projects into an $m_{\Gamma }$-dimensional subspace of $V^{\Gamma }$. For any spherical harmonic basis with sufficiently high angular momentum and a finite group $m_{\Gamma } > 1$. In these cases there are infinitely many ways to construct the subspaces since $\displaystyle \left| \Theta _{m,k}^{\Gamma }\right\rangle$ can rotate freely into $\displaystyle \left| \Theta _{m',k}^{\Gamma }\right\rangle$ [2] .

${\hat{P}}_{kl}^{\Gamma }$ requires the full matrix representations of $\Gamma$ and since the character tables are generated, this would require the representations to be as well. A trace projection operator which can be constructed using only the character tables is therefore used:

$$\begin{aligned} {\hat{P}}^{\Gamma } = \displaystyle \sum _{k}{{\hat{P}}_{kk}^{\Gamma }} = \frac{dim({\Gamma })}{\left| G\right| }\displaystyle \sum _{R \in G}\chi ^{\Gamma }(R){\hat{R}} \end{aligned}$$

**Fig. 5:**
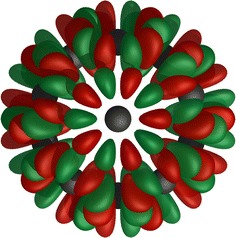
$A_u$ component of $I_{h}$. Visualisation of an $A_u$ component of $I_{h}$ formed by *h* orbitals

This operator projects any function in *W* into $V^{\Gamma }$ but has lost the information required to determine partner functions. $V^{\Gamma }$ can be acquired using Graam-Schmidt or $LDL^T$ decomposition (for real projection operators) of the matrix representation of ${\hat{P}}^{\Gamma }$ in *W* since it is constructed from a linear combination of $\{\left| \Theta ^{\Gamma }\right\rangle \}$. A SALC in $I_h$ where $dim(\Gamma )=1$ is illustrated in Fig. 5.

#### Partner functions

Since the trace projector ${\hat{P}}^{\Gamma }$ cannot separate the components of $V^{\Gamma }$ another method is required in order to generate the subspaces for each component. The spanning vectors for the degenerate spaces also need to transform in corresponding fashion under the point group generators, i.e. they need to be similarly oriented [14], regardless of equivalence set or original basis functions. In order to automate the procedure of obtaining these partner functions, subduction [15] is employed.

Subduction $G\downarrow H$ where *H* is abelian allows for the reduction of $\Gamma$ in *G* into irreps $\gamma$ in *H*. This will split $V^{\Gamma }$ according to:

$$\begin{aligned} V^{\Gamma } = V^{\gamma ^1} + V^{\gamma ^2} + \dots V^{\gamma ^{dim(\Gamma )}} \end{aligned}$$

where $dim(V^{\gamma ^n}) = m_{\Gamma }$ and $V^{\gamma ^n}$ is spanned by $\{\left| \Theta _{*,n}^{\Gamma }\right\rangle \}=\{\left| \Theta ^{\gamma ^n}\right\rangle \}$. An eigenbasis with respect to *H* can subsequently be projected out from $V^{\Gamma }$ and an operator in $G\setminus H$ can used to rotate functions in $V^{\gamma }$ to $V^{\gamma '}$ producing the partner functions according to:

$$\begin{aligned} {\hat{R}}\left| \Theta ^{\gamma ^i}\right\rangle = \displaystyle \sum _{j}{c_j\left| \Theta ^{\gamma ^j}\right\rangle }: R \in G\setminus H \end{aligned}$$

An example of one such subduction for the $C_{3v}$ point group can be seen in Table 2. Since all subgroups $H \in G$ have been determined, all that is required is the choice of *H*. This choice is often arbitrary, but since this work focuses on real spherical harmonics, irreps with complex characters are avoided. In case of 5-dimensional irreps in icosahedral symmetry the subduction is a chain of two steps:

$$\begin{aligned} H \xrightarrow {I \downarrow D_{5}} {\left\{ \begin{array}{ll} A_1 \\ E_1 \xrightarrow {D_{5} \downarrow C_{2}} A^{E_1}+B^{E_1}\\ E_2 \xrightarrow {D_{5} \downarrow C_{2}} A^{E_2}+B^{E_2} \end{array}\right. } \end{aligned}$$

**Fig. 6:**
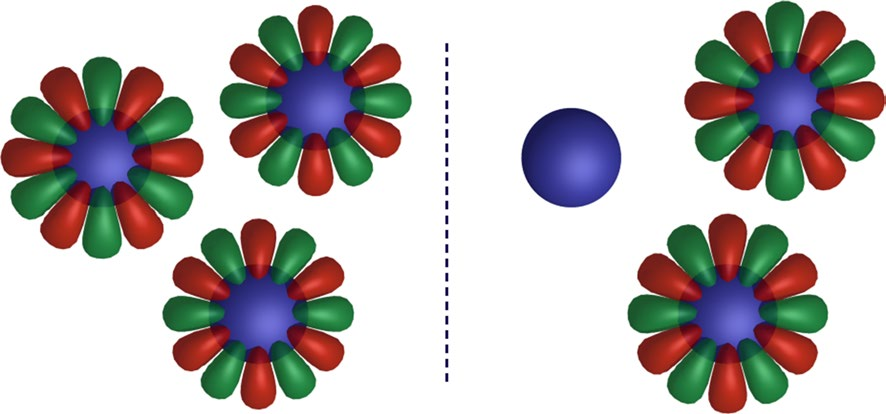
*E* components of $C_{3v}$. Visualisation of the two *E* components of $C_{3v}$ formed by *i* orbitals

The result of on such calculation using *i*-orbitals can be seen in Fig. 6.

#### Direct product representations

One way to construct ${\hat{P}}^{\Gamma }$ is to use the basis of real spherical harmonics on each of the elements $a_i \in {\mathbf {S}}_i^{G}$. There is however some loss of information when using this approach. Rather than constructing one representation we construct two: one from the permutations of ${\mathbf {S}}_i^{G}$ and one from the real spherical harmonics $\{\left\langle Y_{\ell m}\right| \}_{m=-\ell \ldots \ell }$. Since all equivalence sets and spherical harmonics of different angular momentum are treated separately, these bases will simply be denoted *S* and *Y* respectively. The representations can then be constructed using an outer product:

$$\begin{aligned} D^{SY}(R) = D^{S}(R) \otimes D^{Y}(R) \end{aligned}$$

and similarly the space $V^{\Gamma _p,S} \otimes V^{\Gamma _q,Y}$ can be constructed and subsequently separated into the irreps produced by the direct product decomposition of $\Gamma _p \times \Gamma _q$.

#### Matrix representations *D*(*G*)

The $D^{Y}(G)$ and $D^{S}(G)$ matrix representations need to be constructed in order to calculate the projection operator. $D^{Y}(G)$ are calculated using an iterative version of [16], with a sign correction in the $V^l_{mm'}$ function, whereas $D^{S}(G)$ is simply acquired from the permutation information of the nuclei.

#### Linear groups

Linear groups have an infinite number of symmetry operations, and therefore the projection operator cannot be constructed from the representations of the symmetry operations. There are more than one approach to solving this such as aligning the group with the *z*-axis and separating the partner functions based on angular momentum, then applying a transformation to get the actual molecular orientation. The approach used in this work is to reuse the algorithm already present for all other point groups. This requires two additions: (i) a subgroup that will split all functions in the same way as the linear group and (ii) a specially constructed character table. The subgroups which will accomplish this are $C_{(2\ell ) v}$ and $D_{(2\ell ) h}$ for $C_{\infty v}$ and $D_{\infty h}$ respectively. The character tables can then be constructed by assigning the same conjugacy class to the vertical and dihedral ${\hat{C}}_2$ and ${\hat{\sigma }}$ operations for the subgroups.

### Symmetrisation of molecules and wavefunctions

Symmetrisation is achieved using projection, and even though symmetrisation of the nuclei can be done using the same algorithm as for wavefunctions, it can be accomplished using simpler methods.

#### Molecules

Symmetrisation of the nuclei uses projection onto the totally symmetric space. This is a least squares approximation of a line in multi-dimensional space and the component not in the totally symmetric space serves as an error indicator. The basis used is a position vector centred on the nuclei corresponding to the position relative to the centre of mass, similar to that of vibrational modes.

#### Wavefunctions

Wavefunctions are projected onto the space in which it has the larges component. For multi-dimensional irreps the partner functions are determined by constructing and comparing a vector of components for each space. An average component is computed for each irrep, and the orbitals are rotated to align with the symmetry adapted basis functions. This means that the largest components of all wavefunctions must correspond to the span of the symmetry adapted basis functions, i.e. there is a limit to how symmetry broken the input can be.

## Implementation

The procedures described above were implemented in C, with bindings for Python, and the code has been released under an MIT license at https://github.com/mcodev31/libmsym. Various other test implementations were made in other languages, but interoperability with other software written in e.g. Fortran and C++ was a requirement from the start. The library has beem integrated into: the computational code Molcas [17] (to obtain symmetry adapted Hartree-Fock and CASSCF wavefunctions), the molecular modelling softwares Luscus [11], Avogadro^2^ [18] and Avogadro v1 [19]. The Molpy [20] software uses this work to produce an initial set of symmetry adapted orbitals taking the overlap of the basis set into account. The release implementation as of this writing can be found at doi:10.5281/zenodo.245602.

## Conclusion

The symmetry detection algorithms described in this work are capable of detecting the point group of nanotubes as well as large structures such as proteins. The algorithms for generating SALCs and symmetrising molecular orbitals include the generation of vector spaces for irreducible representations, using real spherical harmonics basis functions and determination of partner functions. The algorithm uses direct product decomposition, and splitting fields determined using subgroups information in order to generate a canonical basis for the irreducible representations. The resulting software library can and has been integrated into quantum chemistry software as well as graphical modelling software, and can be used during calculations or for educational purposes.
